# Voltammetric Sensing of Nifedipine Using a Glassy Carbon Electrode Modified with Carbon Nanofibers and Gold Nanoparticles

**DOI:** 10.3390/bios13080829

**Published:** 2023-08-19

**Authors:** Anderson M. Santos, Ademar Wong, Maria H. A. Feitosa, Andy A. Cardenas-Riojas, Sandy L. Calderon-Zavaleta, Angélica M. Baena-Moncada, Maria D. P. T. Sotomayor, Fernando C. Moraes

**Affiliations:** 1 Department of Chemistry, Federal University of São Carlos (UFSCar), São Carlos 13560-970, Brazil; 2 Institute of Chemistry, São Paulo State University (UNESP), Araraquara 14801-970, Brazil; 3 Laboratorio de Investigación de Electroquímica Aplicada, Facultad de Ciencias, Universidad Nacional de Ingeniería, Av. Túpac Amaru 210, Rímac 15333, Peru

**Keywords:** nifedipine, carbon nanofibers, gold nanoparticles, environmental samples

## Abstract

Nifedipine, a widely utilized medication, plays a crucial role in managing blood pressure in humans. Due to its global prevalence and extensive usage, close monitoring is necessary to address this widespread concern effectively. Therefore, the development of an electrochemical sensor based on a glassy carbon electrode modified with carbon nanofibers and gold nanoparticles in a Nafion^®^ film was performed, resulting in an active electrode surface for oxidation of the nifedipine molecule. This was applied, together with a voltammetric methodology, for the analysis of nifedipine in biological and environmental samples, presenting a linear concentration range from 0.020 to 2.5 × 10^−6^ µmol L^−1^ with a limit of detection 2.8 nmol L^−1^. In addition, it presented a good recovery analysis in the complexity of the samples, a low deviation in the presence of interfering potentials, and good repeatability between measurements.

## 1. Introduction

Hypertension is a chronic disease identified by high blood pressure in the arteries, which is detected when the maximum and minimum pressure values are equal to or greater than 140/90 mmHg (14 by 9). An individual identified with this health condition is treated using several strategies, including the use of medication as a viable option to not only help reduce blood pressure but also to avoid other health problems such as heart disease and vascular brain accidents. Thus, drugs such as nifedipine (NIF) are beneficial for controlling blood pressure in humans [1]. Therefore, considering that it is a global problem, nifedipine has become a drug with a potential threat due to possible indiscriminate uses or even living beings that have access to contaminated sewage routes with possible harmful concentrations to the biological system [2].

Of the methods proposed for the detection of NIF in different matrices [3], liquid chromatography [4], UV-vis spectrometry [5], gas chromatography [6], and electrochemical detection are notable [7,8], both in detection and quantification. However, among those cited, chromatographic methods are considered expensive, time-consuming, and require pre-treatment and derivatization steps, not to mention the low sensitivity and selectivity of these methods [9]. In this case, electrochemical techniques positively address most of the figure of merit deficiencies faced by conventional techniques.

Electrochemical detection studies are reported in full. Gaichore and Srivastava [10] developed a nifedipine detection system with a 𝛽-cyclodextrin-modified carbon nanotube paste electrode employing addition stripping voltammetry, where it was 14.8 nmol L^−1^. On the other hand, Baghayeri et al. [11] developed a sensor based on glassy carbon modified with silver nanoparticles, with a detection limit of 0.72 µmol L^−1^. Khairy et al. [12] proposed a sensor using screen-printed electrodes modified with MgO by the differential pulse voltammetry technique, with a detection limit of 32.0 nmol L^−1^. Therefore, it is observed that different catalytic materials, as well as different electrochemical techniques, are excellent tools for documenting the analysis of proposed drugs with promising results.

In recent decades, carbon-based materials have garnered significant attention due to their structural and morphological properties [13,14,15]. Those materials have active centers that generate faradaic and reversible oxidation and reduction reactions originating from interactions between an ionic solution and carbon [16]. Carbon is also highlighted in the area of nanotechnology, which designs ultrafine and nanometric structures to explore reactivity properties, facilitate the diffusion of ions at high speed, and improve the quantum confinement of charges that can be achieved through the morphological control of this material in the form of nanowires [17]. On the other hand, if the use of nanometric carbonaceous materials amplifies the electroanalytical response of a given detection study, the formation of a nanocomposite with these microstructures favorably increases the current response in micro-analytical analyses. For example, nanoparticles of noble materials (gold, platinum, and silver) are of great prominence in the field of electroanalysis focused on environmental analysis [18,19,20], as gold nanospheres have a diameter between 1 and 100 nm that contributes to high surface energy and their surface ratio–volume favors the immobilization of a large number of molecules that suppress their catalytic activity [21]. Finally, when this class of materials is used for the oxidation of electroactive species in a sensing application, they provide a direct and rapid electron transfer between a variety of species capable of oxidation or reduction reactions [18,22].

In this work, the use of a carbon nanofiber film modified with gold nanoparticles and Nafion^®^ for the subsequent modification of the glassy carbon electrode (AuNPs:CNF-NF/GCE) aims at the determination of NIF in biological and environmental samples. The advantage of this method lies in the preparation of the sensor, as AuNPs are synthesized directly in the CNF, eliminating the need for any further steps to prepare the dispersion and/or modification of the GCE, bearing in mind that most works report the electrodeposition of AuNPs.

## 2. Materials and Methods

### 2.1. Reagents and Solutions

Nifedipine (NIF), Nafion^®^ (NF), gold (III) acid chloride trihydrate (HAuCl_4_.3H_2_O), carbon nanofibers (CNF), sodium borohydride (NaBH_4_), and bovine serum were purchased from Sigma-Aldrich. The reagents used in the preparation of the electrolyte, such as H_3_PO_4_, KH_2_PO_4_, K_2_HPO_4_, K_3_PO_4_, CH_3_COONa, and CH_3_COOH, were purchased from Acros. In this context, all reagents used were of analytical grade with purity ≥ 98%. Furthermore, all solutions were prepared with ultrapure water (resistivity ≥ 18.0 MΩ cm) obtained by a Milli-Q Direct 8 purification system (Millipore^®^, Billerica, MA, USA).

### 2.2. Apparatus

All electrochemical analyses were performed using an Autolab model PGSTAT12 potentiostat/galvanostat controlled by the Nova 2.1 software. Voltammetric measures were performed using an electrochemical cell (10.0 mL) with three electrodes: an Ag/AgCl/3.0 mol L^−1^ KCl as a reference electrode, a platinum sheet (0.50 cm^2^) as a counter electrode, and a glassy carbon electrode (GCE, Ø = 3.0 mm) as a working electrode. The morphological characterization of the nanomaterials (CNF and AuNPs:CNF) used in the GCE modification was performed by field emission gun scanning electron microscopy (FEG/SEM, FEI Magellan 400L).

### 2.3. CNF Activation and CNF Modification with Gold Nanoparticles

The CNF were chemically activated using a mixture of concentrated acids (H_2_SO_4_/HNO_3_: *v*/*v*) for approximately 12 h under stirring. Subsequently, the material was washed to a pH close to 7.0 and dried in an oven. Next, AuNPs:CNF material was synthesized as proposed by Cardenas-Riojas et al. [23]. For this, 100 mg of CNF were mixed with 80 mL of gold salt (HAuCl_4_.3H_2_O) 0.010 µmol L^−1^ at pH 9.5 (pH adjusted with NaOH). Thus, for the formation of AuNPs, 1.0 mL of NaBH_4_ 0.10 mol L^−1^ solution was added at room temperature for the formation and growth of AuNPs on the surface of CNF. In sequence, the obtained solution was heated to remove unreacted borohydride ions, and subsequently, the obtained composite was centrifuged 4 times at 10,000 rpm in a NaOH solution (pH 9.5). Finally, the drying procedure was performed in an oven at 60 °C for approximately 24 h. Thus, obtaining the composite CNF:AuNPs.

### 2.4. Preparation of the Modified GCE

Initially, before modification, the surface of the GCE was carefully cleaned. For this, the electrode surface was polished using alumina microparticles (0.5 µm) and a polishing cloth, followed by ultrasonic cleaning with isopropyl alcohol and ultrapure water for 1 min each. The CNF-NF or AuNPs:CNF-NF dispersion was prepared with 1.0 mg of the material (CNF or AuNPs:CNF), 50 µL of 0.50% (*v*/*v*) NF solution, and 950 µL of ultrapure water. The mixture obtained was subjected to ultrasonic stirring for 60 min. Then, an 8.0 µL aliquot of the freshly prepared dispersion was deposited on the GCE surface and dried in a desiccator (25 °C) for 2 h, obtaining the GCE modified with CNF-NF or AuNPs:CNF-NF film.

### 2.5. Preparation of the Samples

The synthetic urine sample was prepared as described in the literature [24]. In a volumetric flask (50 mL), 0.40 mmol L^−1^ KCl, 0.20 mmol L^−1^ NaCl, 0.30 mmol L^−1^ KH_2_PO_4_, 0.20 mmol L^−1^ CaCl_2_, 0.36 mmol L^−1^ NH_4_Cl, and 0.36 mmol L^−1^ urea were added. However, the serum sample used was purchased commercially without any type of treatment. In both cases, each sample (synthetic urine and serum) was separated into two flasks and doped with two known concentrations of NIF. For the analysis, 300 µL of each sample were added separately to the electrochemical cell (final concentration: 0.40 and 1.0 μmol L^−1^) and individually analyzed using the addition and recovery method.

## 3. Results and Discussion

### 3.1. Morphological and Chemical Characterization

**Figure 1:**
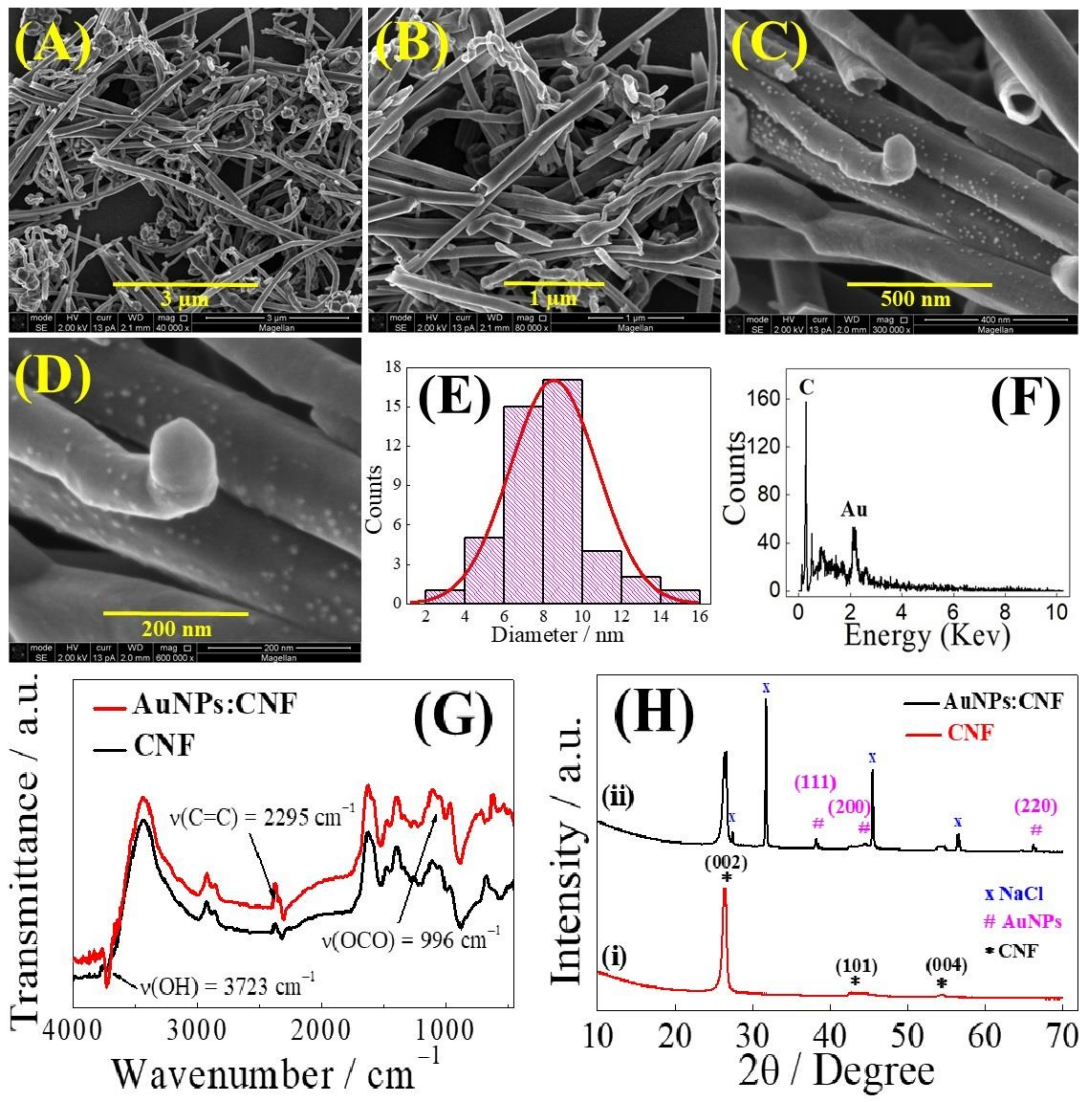
SEM (**A**,**B**) CNF, and (**C**,**D**) AuNPs:CNF in different magnifications. (**E**) Corresponding histogram of AuNPs diameters. (**F**) Corresponding EDX spectrum of the AuNPs:CNF. (**G**) IR spectrum of CNF and AuNPs:CNF materials. (**H**) XRD pattern of CNF and AuNPs:CNF.

Morphological characterization of CNF and AuNPs:CNF was obtained by scanning electron microscopy (SEM). As seen in Figure 1A,B at different magnitudes, the cylindrical shape of the CNF showed a uniform distribution with varied diameters and lengths. On the other hand, as can be seen in Figure 1C,D, the AuNPs:CNF material showed the same cylindrical shape as the carbon nanofibers but with numerous distributed spherical nanoparticles, showing that there was the incorporation of the AuNPs. The mean diameter of nanoparticles was estimated using ImageJ software. In this way, it was possible to obtain an average value of AuNPs of 8.5 nm (Figure 1E).

The chemical characterization of the materials was performed using an energy-dispersive X-ray detector (EDX) and infrared spectrum. As can be observed in Figure 1F, the EDX spectrum showed the presence of carbon atoms belonging to CNF and gold atoms belonging to AuNPs, as expected. The infrared spectrum of CNF and AuNPs:CNF is presented in Figure 1G, and characteristic vibrations of the C-OH, C=C, and OCO bonds at 3723, 2295, and 996 cm^−1^, respectively, are observed, which are typical of the carbonaceous material CNF. Then, when the surface of the CNF is modified with the gold nanoparticles, the same vibrations of these links are observed, but with greater intensity; this is due to the presence of this nanomaterial, which indicates that the surface of the CNF was modified by the in situ synthesis of AuNPs and the synergy between them, which will allow us to use them as material for detection [25,26].

Finally, X-ray diffraction (XRD) analysis was used to characterize the properties of CNF and AuNPs:CNF materials, as can be seen in the diffractograms in Figure 1H. For the CNF (diffractogram (i)), it is possible to observe three peaks, an intense peak at 26.4° and two weak peaks, one at 43.3° and another at 54.4°, referring to the graphite planes (002), (101), and (004), respectively, according to the Joint Committee on Powder Diffraction Standards (JCPDS 75-1621). On the other hand, when analyzing the AuNPs:CNF material (diffractogram (ii)), it is possible to observe three diffraction peaks at scattering angles of 38.3°, 44.5°, and 64.4°, corresponding to the crystal planes (111), (200), and (220), respectively, which is indicative of the presence of AuNPs (JCPDS 04-0784). Furthermore, the other diffraction peaks (27.5°, 31.7°, 45.4°, and 56.5 refer to NaCl (JCPDS 06-0528), which, in turn, was obtained during the synthesis of AuNPs. Based on the above results, we can conclude that the AuNPs:CNF material was successfully prepared.

### 3.2. Electrochemical Characterization of Electrodes

**Figure 2:**
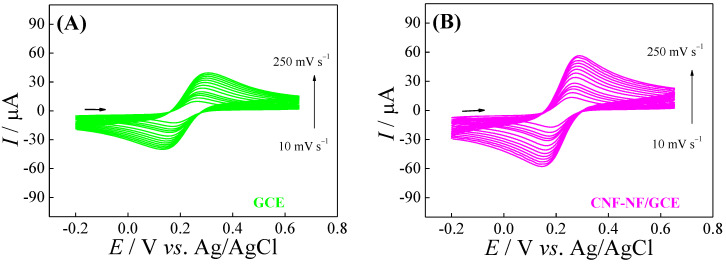

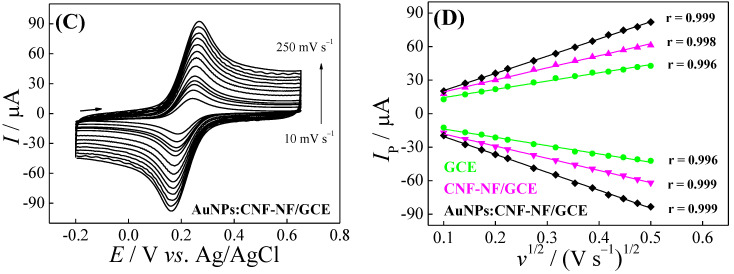
Cyclic voltammograms for a 2.5 mmol L^−1^ [Fe(CN)_6_]^3–^ in 0.10 mol L^−1^ KCl solution using (**A**) GCE, (**B**) CNF-NF/GCE, and (**C**) AuNPs:CNF-NF/GCE at different potential scan rates (10–250 mV s^−1^). (**D**) Graphics of *I*_p_ values in function of the square root of the potential scan rate (*v*^1/2^). The arrows indicate the scan direction.

The electroactive areas of GCE, CNF-NF/GCE, and AuNPs:CNF-NF/GCE were estimated using the Randles–Sevcik equation (Equation (1)) [27]. For this, cyclic voltammetry (CV) was performed using the redox probe [Fe(CN)_6_]^3−^ (2.5 mmol L^−1^) at different potential scan rates (10 − 250 mV s^−1^) in order to effectively evaluate the electrochemical response of each electrode (Figure 2).

*I*_p_ = ±(2.69 × 10^5^) *n*^3/2^
*A D*^1/2^
*C v*^1/2^, (1)

where *I*_p_, *A*, *n*, *D*, *v*, and *C* are the peak current (A), electroactive area (cm^2^), number of electrons transferred, the diffusion coefficient of [Fe(CN)6]^3–^ (7.6 µcm^2^ s^−1^), the potential scan rate (V s^−1^), and the concentration of [Fe(CN)_6_]^3–^ (mol cm^−3^), respectively.

The calculated electroactive areas were 0.052, 0.076, and 0.11 cm^2^ for GCE, CNF-NF/GCE, and AuNPs:CNF-NF/GCE, respectively. From the results obtained with the electrodes, it can be observed that the modification of the surface of the electrodes increased the electroactive area by two times when comparing the results of the AuNPs:CNF-NF/GCE electrode in relation to the bare GCE and 1.4 times for the CNF-NF/GC.

Finally, in order to verify the kinetic process of charge transfer promoted by the modification of the electrode surface, the constant *k*^0^ (heterogeneous electron transfer rate constant) was calculated using the Nicholson equation [28] (Equation (2)):

Ψ = k^0^ [π D n v F/(R T)]^−1/2^, (2)

where Ψ is the kinetic parameter, *T* is the absolute temperature (298 K), and π, *F*, and *R* are the mathematical (3.1415), Faraday (96485 C mol^−1^), and universal gas (8.314 J K^−1^ mol^−1^) constants, respectively.

For this, the values of Ψ were obtained using Equation (3) (for *n* × Δ*E*_p_ < 200 mV) proposed by Lavagnini et al. [29]:

Ψ = (−0.6288 + 0.0021nΔE_p_)/(1 − 0.017nΔE_p_). (3)

Therefore, when using these equations, the *k*^0^ values are acquired by the angular coefficients (slopes) of the resulting curves: 2.8 mcm s^−1^ for GCE, 4.8 mcm s^−1^ for CNF-NF/GCE, and 18.0 mcm s^−1^ for AuNPs:CNF-NF/GCE. Therefore, an increase in the value of *k*^0^ indicates how quickly the system enters equilibrium driven by the modification of the electrode surface. That is, the *k*^0^ values are a measure of the kinetic ease of the redox couple.

### 3.3. Electrochemical Behavior of NIF

**Figure 3:**
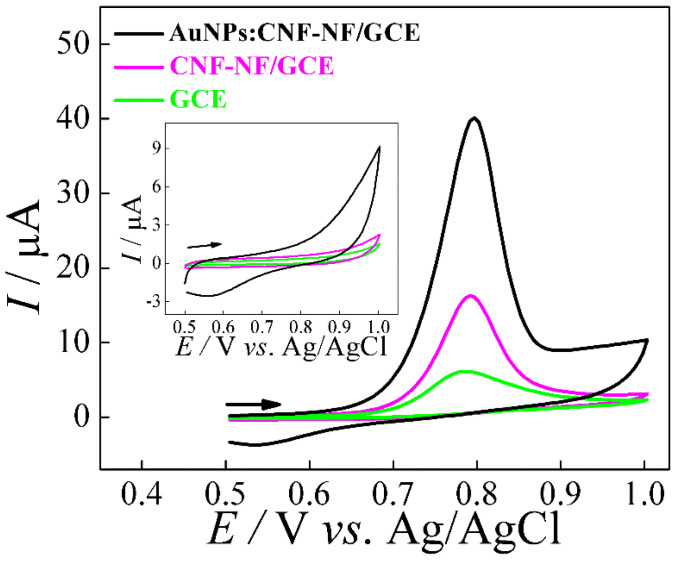
VC obtained using a GCE (–), CNF-NF/GCE (–), and AuNPs:CNF-NF/GCE (–) in the presence (0.10 mmol L^−1^) and absence (inserted) of NIF in 0.10 mol L^−1^ phosphate buffer (pH 7.0). *v* = 50 mV s^−1^; *t*_acc_ = 20 s. The arrows indicate the scan direction.

The voltammetric profile of the NIF molecule using the electrodes GCE, CNF-NF/GCE, and AuNPs:CNF-NF/GCE was evaluated by CV in the potential range of 0.50–1.0 V (*v* = 50 mV s^−1^). As seen in Figure 3, the voltammetric profile for NIF showed only a well-defined oxidation peak at the 0.80 V potential, i.e., indicating an irreversible process, as it does not show any reduction peak in the studied range.

Furthermore, it is possible to notice that there was an increase in the magnitude of the peak current (*I*_p_) during the electrode modification. When only GCE was used, there was a response of *I*_p_ = 5.90 µA. When GCE was modified with CNF, an increase in *I*_p_ to 16.5 µA was observed, and when AuNPs were incorporated into the carbonaceous material (CNF), an even greater increase in *I*_p_ to 40.3 µA was observed (Figure 3). This modification with AuNPs:CNF on the GCE surface contributed to a 6.8-fold increase in NIF detection.

The increase in electroanalytical response seen in the voltammetric profile is due to the morphological characteristic of the material, which contributes to adding greater porosity and conductivity to the surface of the electrode (GCE), which provides a better drug detection response concerning the smoother surface of the clean GCE. Thereby indicating that GCE modified with AuNPs:CNF is essential to achieve lower NIF concentration values.

### 3.4. Mechanism Proposal of NIF Electrochemical Oxidation

A mechanism proposal for NIF electrochemical oxidation was performed using a study of the pH effect and scan rate studies.

**Figure 4:**
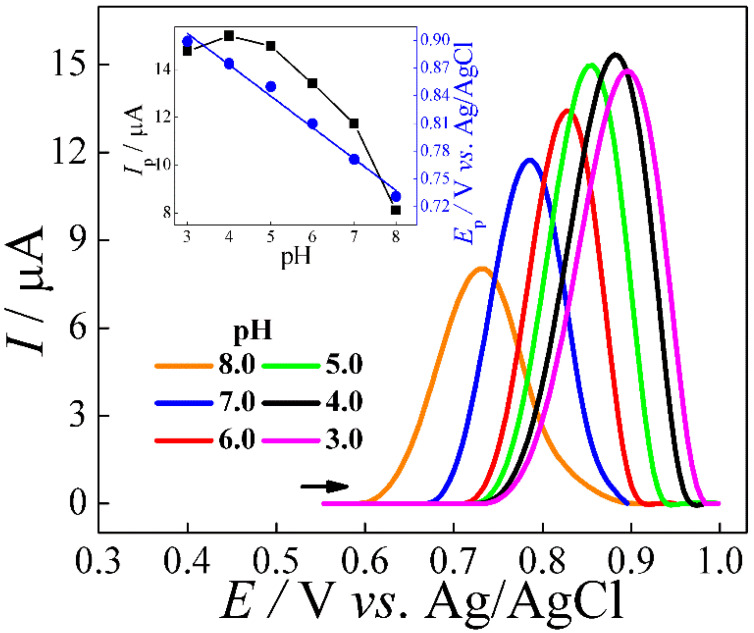
SW voltammograms recorded for 2.0 µmol L^−1^ NIF using 0.10 mol L^−1^ phosphate-buffered solution with pH ranging from 3.0 to 8.0 using the AuNPs:CNF-NF/GCE sensor. Parameters: *f* = 15 Hz, *a* = 40 mV, Δ*E*_s_ = 5 mV s^−1^, and *t*_acc_ = 20 s. Insert: *I*_p_ vs. pH and *E*_p_ vs. pH. The arrows indicate the scan direction.

Square-wave voltammetry (SWV) was performed to discover the pH (from 3.0 to 8.0) effect on the NIF (2.0 µmol L^−1^) oxidation reaction. As observed in Figure 4, the optimal pH of the electrolyte is 4.0, at which the highest magnitude of peak current was obtained.

For this reason, pH 4.0 was selected for the rest of the studies. Furthermore, as can be seen in Figure 4 (inset graph), with the dependence of peak potential (*E*_p_) versus pH (*E*_p_ vs. pH), with increasing pH, there is a change in peak potential to less positive values indicating the participation of proton transfer in the reaction. The slope of *E*_p_ vs. pH is 43 mV, of which the ratio of 1 (*m*/*n*) is estimated, as previously reported by Gaichore et al. [10].

**Figure 5:**
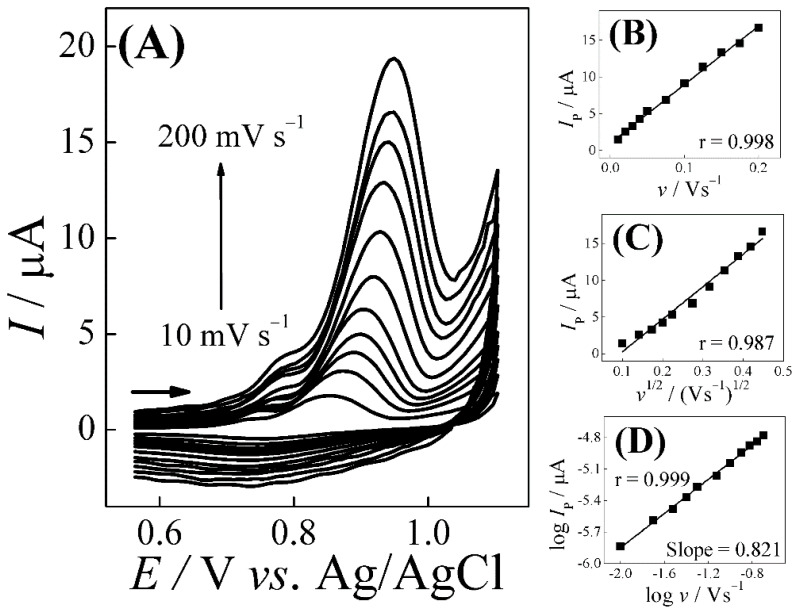
(**A**) Cyclic voltammograms for different scan rates (10–200 mV s^−1^) using the AuNPs:CNF-NF/GCE sensor for 50.0 µmol L^−1^ NIF in 0.10 mol L^−1^ phosphate-buffered solution (pH 4.0). Graphics of (**B**) *I*_p_ vs. *v*, (**C**) *I*_p_ vs. *v*^1/2^, and (**D**) log *I*_p_ vs. log *v*. The arrows indicate the scan direction.

The effect of the potential scan rate was performed using CV, varying from 10 to 200 mV s^−1^ in the presence of 50.0 µmol L^−1^ NIF. As seen in Figure 5A, with increasing potential scan rate, there is a change in potential to more positive values (characteristic of irreversible reactions) along with a gradual increase in the magnitude of the peak current. Furthermore, the results showed good linearity in the relationship of Δ*I*_pa_ vs. *v* (Figure 5B) when compared with Δ*I*_pa_ vs. *v*^1/2^ (Figure 5C), indicating that NIF oxidation is controlled by adsorption on AuNPs:CNF-NF/GCE. This process is proved by the relationship between log Δ*I*_p_ vs. log *v* (Figure 5D), which presented a slope of 0.821, closer to the theoretical value of 1.0, and characteristic of an adsorption-controlled system.

**Scheme 1:**
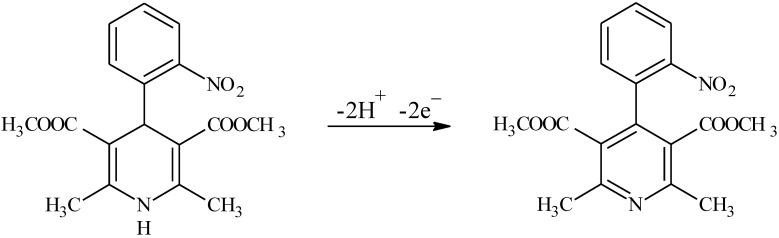
Possible reaction for NIF electro-oxidation [10].

Next, to elucidate the reaction mechanism, the number of electrons (*ɳ*_e_) involved in the NIF electro-oxidation reaction was calculated from the study using CV at 50 mV s^−1^, according to Equation (4).

(4) $$E_{p}-E_{p/2}=\frac{47.7}{\left(\alpha \times {ɳ}_{e}\right)}\;\mathrm{mV},$$

where *E*_p_ = 0.903, *E*_p/2_ = 0.853, and the electron transfer coefficient (α) is equal to 0.5 (completely irreversible reaction) [27]. Substituting the values into the equation, we see that the value of *ɳ*_e_ is 1.91, which is close to 2. Considering the *m*/*n* value of 1 (previously obtained), an equal number of electrons and protons (2e^−^ and 2H^+^) are involved in the electro-oxidation reaction of the NIF molecule. The same electrochemical behavior has been reported in other studies [10,12,30]. The probable reaction for NIF oxidation, as previously reported in the literature, is shown in Scheme 1.

### 3.5. Optimization of SWV Parameters

The analytical parameters that affected the NIF response using the SWV technique, such as amplitude (*a*), frequency (*f*), and potential increment (Δ*E*_s_), were optimized (see Table S1), and the optimal values are *a* = 60 mV, *f* = 30 Hz, and Δ*E*_s_ = 7 mV. Furthermore, the application of pre-concentration potentials (−0.2 to 0.8 V) and the agitation/accumulation time (*t*_acc_) (10 to 50 s) were also evaluated. However, no significant increase was observed applying pre-concentration potentials. Therefore, an open potential (without applying a fixed potential) and *t*_acc_ 30 s were used.

### 3.6. Voltammetric Determination of Nifedipine

**Figure 6:**
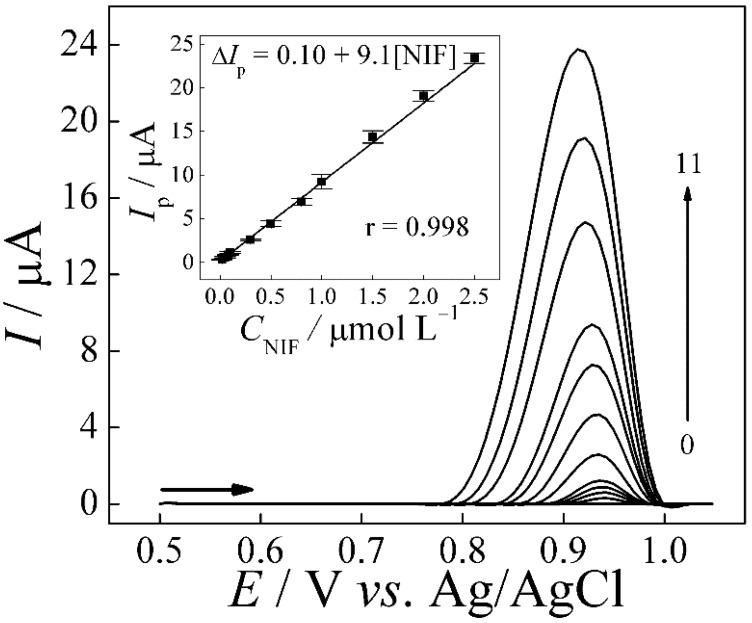
SW voltammograms obtained using the AuNPs:CNF-NF/GCE sensor in 0.10 mol L^−1^ phosphate solution (pH 4.0) containing different concentrations for NIF: (0–11) 0, 0.020, 0.050, 0.080, 0.10, 0.30, 0.50, 0.80, 1.0, 1.5, 2.0, and 2.5 µmol L^−1^. Insert: analytical curve. SWV parameters: *a* = 60 mV, *f* = 30 Hz, Δ*E*_s_ = 7 mV, and *t*_acc_ = 30 s. The arrows indicate the scan direction.

After the entire process of optimizing the SWV analytical parameters, the AuNPs:CNF-NF/GCE sensor was used to explore the electrochemical response of the NIF as a function of concentration variation. Figure 6 shows that the calibration curve (variation of peak current magnitude versus concentration variation) for the NIF presented a linear analytical response (r = 0.999) in the concentration range of 0.020–2.5 µmol L^−1^ with the following linear regression calibration curve (Equation (5)):

*I*_p_ (µA) = 0.10 + 9.1 [NIF] (µmol L^−1^) r = 0.998. (5)

Then, the limit of detection (LOD) and quantification (LOQ) were determined according to the criteria: LOD = “3σ”/“*m*” and LOQ = “10σ”/“*m*”, where “σ” is the deviation standard of ten measurements of the blank (supporting electrolyte only) and “*m*” is the slope of the analytical curve. Thus, the values obtained were 2.8 nmol L^−1^ and 9.4 nmol L^−1^ for LOD and LOQ, respectively.

The analytical performance obtained by the proposed voltammetric method using the AuNPs:CNF-NF/GCE sensor was compared with other NIF voltammetric determination methods already reported in the literature, as shown in Table 1 [1,10,11,12,31,32]. In this context, the detection method presented here shows the best working set of working results in terms of sensitivity and/or linear concentration range. GCE modified with AuNPs:CNF-NF highlights high selectivity and stability, easy sensor preparation (electrode modification), excellent repeatability, and reproducibility of the modification, as presented below.

### 3.7. Study of Repeatability, Reproducibility, and Selectivity

Repeatability, reproducibility, and selectivity studies of the AuNPs:CNF-NF/GCE sensor were evaluated using concentrations of 1.0 µmol L^−1^ NIF in a 0.10 mol L^−1^ phosphate-buffered solution (pH 4.0). For the repeatability study (Figure S1A), 20 measurements (*n* = 20) were performed intra-day using the same sensor, and for the reproducibility study of modification (Figure S1B), four distinct glassy carbon electrodes (GCE) were modified using the same dispersion (AuNPs:CNF-NF) and analyzed separately (*n* = 3). Accordingly, the RSD values obtained for repeatability analysis are 5.61%, and for reproducibility analysis, the procedure presented an RSD value of 2.28%. The low RSD values obtained indicate that the proposed analytical method has an excellent response of repeatability and reproducibility using the sensor in NIF detection.

Subsequently, the proposed sensor selectivity study was performed in the presence of different potential interferents (ascorbic acid, atenolol, caffeine, dopamine, uric acid, urea, Cd^2+^, Pb^2+^, Cu^2+^, Zn^2+^, and humic acid) in the concentration ratio of 1:10 (analyte:interfering potential). As can be observed in Table S2 (electronic supply material), the results obtained show an error between −1.1% and 2.3% in all cases, indicating that the sensor AuNPs:CNF-NF/GCE can be applied efficiently to the determination of the NIF in the presence of these possible interferents to the concentration analyzed.

### 3.8. Applications in Biological and Environmental Samples

Finally, to evaluate the electroanalytical method developed, NIF was determined in serum, synthetic urine, and river water samples. For this, the different samples were doped with two concentrations (0.100 and 1.00 µmol L^−1^) of NIF, analyzed, and calculated by the interpolation method of the calibration curve. As can be seen in Table 2, the recovery percentages obtained in all samples were satisfactory, ranging from 95.7% to 108%, demonstrating that the proposed method can be effectively applied in the determination of NIF in different matrices.

## 4. Conclusions

The excellent electrochemical results obtained with the AuNPs:CNF-NF/GCE sensor were only possible due to the modification of the GCE with AuNPs:CNF, which resulted in an improvement in the analytical signal with the increase in the magnitude of the peak current for the determination of the NIF molecule. These results indicate that carbon nanofibers significantly increase the drug oxidation response compared to the glassy carbon electrode, and when this was decorated with gold nanospheres, the response improved rapidly by 6.8 times, confirming that the nanometric morphology of the carbonaceous materials is quite expressive when compared with the same material class in different formats. Furthermore, it presented a very low limit of detection (2.8 nmol L^−1^) and quantification (9.4 nmol L^−1^), which includes detections of this drug at trace concentration levels similar to those found in effluent treatment plants, rivers, or lakes. The proposed method proved to be stable, reproducible, and effective for the determination of NIF in serum and urine samples. Thus, the methodology developed proved to be highly reliable and advantageous for this type of analysis.

## Figures and Tables

**Table 1:** Analytical parameters comparison obtained for the AuNPs:CNF-NF/GCE sensor and other sensors reported for the determination of NIF.

Electrode	Technique	Linear Range (µmol L^−1^)	LOD (nmol L^−1^)	Matrice	Ref.
βCD-CNT-PE	DPAdSV	0.0477–20.0	14.7	Tablet, urine and serum	[10]
AgNPs/GCE	DPV	0.80–60	720	Tablet and urine	[11]
MgO-SPEs	DPV	0.20–104	32	Tablet and urine	[12]
GRs–IL–hPdAg/GCE	DPV	0.010–4.0	4.0	Tablet	[31]
BDD	DPV	3.98–107	612	Tablet	[1]
Polyfurfural film/GCE	DPV	0.010–7.0	5.0	Tablet and urine	[32]
AuNPs:CNF-NF/GCE	SWV	0.020–2.5	2.8	River water, urine and serum	This work

SPEs: screen-printed electrodes, βCD: β-cyclodextrin, CNT: carbon nanotube, PE: paste electrode, GRs: graphene nanoribbons, IL: ionic liquid, hPdAg: hollow PdAg alloy nanoparticles, BDD: boron-doped diamond.

**Table 2:** Results obtained from the analysis of serum, urine, and river water samples.

Samples	Added (µmol L^−1^)	Found (µmol L^−1^) *	Recovery (%) **
Serum A	0.100	0.101 ± 0.005	101
Serum B	1.00	1.05 ± 0.11	105
Urine A	0.100	0.103 ± 0.005	103
Urine B	1.00	0.960 ± 0.008	95.7
River water A	0.100	0.106 ± 0.009	106
River water B	1.00	1.08 ± 0.05	108

* Average of 3 measured concentrations; ** Recovery percentage = [Found/Added] × 100.

## Data Availability

Not applicable.

## Supplementary Materials

The following supporting information can be downloaded at: https://www.mdpi.com/article/10.3390/bios13080829/s1. Figure S1: Analysis of the repeatability and reproducibility of the AuNPs:CNF-NF/GCE sensor in the presence of 1.0 μmol L^−1^ NIF and phosphate buffer pH 4.0. (A) During 20 measurements performed on the same day and using the same electrode and (B) using four different electrodes manufactured on the same day. SWV parameters: *a* = 60 mV, *f* = 30 Hz, Δ*E*_s_ = 7 mV, and *t*_acc_ = 30 s. Table S1: Optimized analytical parameters of square wave voltammetry (SWV). Table S2: Effect of possible interferents on the SW voltammetric in 0.10 mol L^−1^ phosphate buffer (pH 4.0) solution using an AuNPs:CNF-NF/GCE sensor.
