# Supplementary material for: Voltammetric Sensing of Nifedipine Using a Glassy Carbon Electrode Modified with Carbon Nanofibers and Gold Nanoparticles
Source: Biosensors (Basel). 2023 Aug 19;13(8):829. doi: 10.3390/bios13080829 (PMC10452116; doi:10.3390/bios13080829)
Supplement: Supplementary file 1 [file biosensors-13-00829-s001.zip › biosensors-2566233-supplementary.pdf]

Supplementary Material

Voltammetric Sensing of Nifedipine Using a Glassy Carbon Electrode Modified with Carbon Nanofibers and Gold Nanoparticles

Anderson M. Santos [1], Ademar Wong [2], Maria H. A. Feitosa [1], Andy A. Cardenas-Riojas [3], Sandy L. Calderon-Zavaleta [3], Angélica M. Baena-Moncada [3], Maria D. P. T. Sotomayor [2] and Fernando C. Moraes [1,*]

[1] Department of Chemistry, Federal University of São Carlos (UFSCar), São Carlos 13560-970, Brazil
[2] Institute of Chemistry, São Paulo State University (UNESP), Araraquara 14801-970, Brazil
[3] Laboratorio de Investigación de Electroquímica Aplicada, Facultad de Ciencias, Universidad Nacional de Ingeniería, Av. Túpac Amaru 210, Rímac 15333, Peru
* Correspondence: fcmoraes@ufscar.br

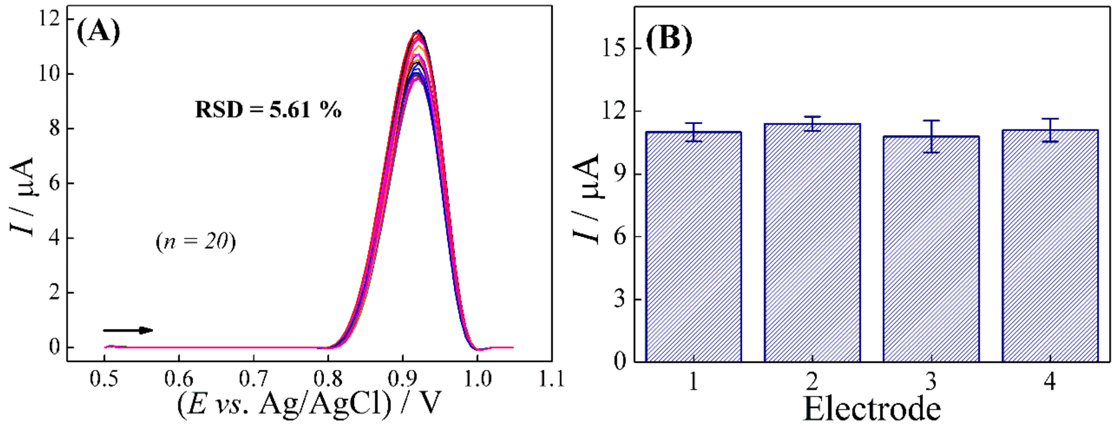

Figure S1. Analysis of the repeatability and reproducibility of the AuNPs:CNF-NF/GCE sensor in the presence of 1.0 μmol L^{-1} NIF and phosphate buffer pH 4.0. **(A)** During 20 measurements performed on the same day and using the same electrode and **(B)** using four different electrodes manufactured on the same day. SWV parameters: a = 60 mV, f = 30 Hz, ΔE_s = 7 mV, and t_{acc} = 30 s.

Table S1: Optimized analytical parameters of square wave voltammetry (SWV)

Parameter	Range	Optimal value
Frequency (Hz)	8 – 35	30
Amplitude (mV)	10 – 100	60
Step (mV)	2 – 9	7
Pre-concentration potential (V)	-0.2 – 0.8	--
Accumulation time, t_{acc} (s)	10 – 50	30

Table S2: Effect of possible interferents on the SW voltammetric in 0.10 mol L^{-1} phosphate buffer (pH 4.0) solution using an AuNPs:CNF-NF/GCE sensor.

Possible interferents	Error (%)* (1 : 10)
Ascorbic acid	−0.6
Atenolol	1.5
Caffeine	0.9
Dopamine	1.3
Uric acid	−1.1
Urea	1.7
Cd^{2+}, Pb^{2+}, Cu^{2+} and Zn^{2+}	0.6
Humic acid	2.3

*Error = [(Analytical $signal_{presence}$ − Analytical $signal_{absence}$) / Analytical $signal_{absence}$] × 100 %.
